# Circulating interleukin-38 concentrations in healthy adults

**DOI:** 10.3389/fimmu.2022.964365

**Published:** 2022-08-09

**Authors:** Lisa U. Teufel, Dennis M. de Graaf, Mihai G. Netea, Charles A. Dinarello, Leo A. B. Joosten, Rob J. W. Arts

**Affiliations:** ^1^ Department of Internal Medicine, Radboud Institute of Molecular Life Sciences (RIMLS) and Radboudumc Center for Infectious Diseases (RCI), Radboud University Medical Center, Nijmegen, Netherlands; ^2^ Department of Medicine, University of Colorado, Aurora, CO, United States; ^3^ Institute of Innate Immunity, University Hospital, University of Bonn, Bonn, Germany; ^4^ Department of Immunology and Metabolism, Life and Medical Sciences Institute, University of Bonn, Bonn, Germany; ^5^ Department of Medical Genetics, Iuliu Hatieganu University of Medicine and Pharmacy, Cluj-Napoca, Romania

**Keywords:** interleukin 38, cytokines, immunity, reference range, healthy adults

## Abstract

Interleukin (IL)-38 is the latest discovered member of the interleukin-1 family, which has anti-inflammatory properties similar to IL-36Ra. Several studies compared circulating IL-38 concentrations in healthy and diseased populations to characterize its role in both auto-immune and inflammatory pathologies, with both higher and lower concentrations being associated with certain diseases. However, in order to use IL-38 as a biomarker, a reference range in healthy adults is needed. To establish a reference IL-38 circulating concentration, accessible data from 25 eligible studies with IL-38 concentrations in healthy adults was collected. To validate the values found in literature, we measured IL-38 concentrations by enzyme-linked immunosorbent assay (ELISA) in several cohorts from our own institute. Additionally, the effect of blood collection techniques, freeze thawing cycles, and hemolysis on IL-38 measurements was assessed. To evaluate the importance of the genetic background of individuals as confounding factor of IL-38 synthesis, we used publicly available eQTL databases with matched data on allele frequencies in individuals of different ethnicities. Mean IL-38 concentrations in the various studies were weighted by their corresponding sample size, resulting in a weighted mean, and weighted upper and lower limits were calculated by mean ± 2 SD. Differences of over 10.000-fold were found in the weighted means between studies, which could not be attributed to the blood collection method or assessment of IL-38 in plasma or serum. Although IL-38 concentrations were markedly higher in Chinese then in European population studies, we could not show an association with the genetic background. From our analysis, a reference range for circulating IL-38 in healthy adults could thus not yet be established.

## Introduction

Interleukin (IL)-38 is a member of the interleukin-1 family with anti-inflammatory properties. It shares, respectively, a 41% and 43% sequence homology with IL-1 receptor antagonist and IL-36 receptor antagonist (1). IL-38 binds to the IL-36 receptor (synonym IL-1R6 or *IL1RL2*) (2) and to TIGIRR-2 (synonym IL-1R9 or *IL1RAPL1*) (3) as a putative (co-)receptor. Apart from these primary receptors, IL-38 is also able to bind to IL-1R1, although with lower affinity than IL-1β or IL-1Ra (3). IL-38 lacks a specific caspase-1 cleavage site and N-terminal processing is required for its biological activation (3, 4). Cleaved IL-38 attenuates the activation of JNK and Activator Protein (AP)-1 signaling, resulting in lower IL-6 production by macrophages upon IL-1β stimulation and reduced differentiation of lymphocytes into Th17 lymphocytes (3). Furthermore, it has been shown that IL-38 reduces mTOR activation and consequently pro-inflammatory cytokine production, as well as the induction of trained immunity by β-glucan in a murine model (5).

With regard to its role in health and disease, aberrant IL-38 concentrations have been observed in different types of diseases such as auto-immune diseases, infections, or acute cardiovascular events (6, 7). Evidence of IL-38’s acute role in response to local or systemic infections, however, is scarce. Some studies considered IL-38 as a disease biomarker, e.g. discriminating low and high disease activity, or predicting disease progression or response to therapy. However, in order to use IL-38 as a biomarker in disease, reference concentrations in healthy adults must be available to determine a discriminatory value. To the best of our knowledge, no such reference has been established for IL-38, as each of the aforementioned studies has used its own (matched) control group to determine a physiological reference circulating concentration.

Only very few studies have been performed to establish the physiological ranges for cytokine or chemokine concentrations in order to use them as diagnostic or prognostic biomarkers (8–10). Although some groups have assessed the IL-1 family specifically, IL-38 was not included in these analyses (11). In 2013, a comprehensive overview of several cytokine and chemokine concentrations in 72 healthy adults was published, however, also here IL-38 was not part of study (12). Recently, a similar study established a reference range of IL-37 circulating concentrations in healthy adults based on studies from literature (13).

The aim of this current study is to collect all available data from literature on IL-38 circulating concentrations in healthy adults to establish a reference range. Apart from these published data, we added several cohorts from our own institute for validation in which IL-38 concentrations were recently determined. Lastly, we investigated the effect of potentially confounding factors on the quantification of IL-38 concentrations such as blood collection tube, freeze thawing cycles, and hemolysis.

## Methods

### Search strategy

A literature search was performed on PubMed for articles published up to the 1st of December 2021 that contained data on blood IL-38 concentrations in healthy human adults using the following search string: IL38 OR IL-38 OR “IL 38” OR interleukin-38 OR “interleukin 38”. Search results were filtered for English, Dutch, German, and French languages.

### Article screening and selection

All available full articles were screened. For inclusion into the analysis, studies had to report serum or plasma concentrations of IL-38 from healthy adults. IL-38 concentrations had to be reported as mean with standard deviation or standard error of the mean, or as range. The included articles were additionally screened for relevant references (snowballing) which were also included if not yet present in the primary search. Papers were excluded if they met any of the following exclusion conditions: i) no quantified protein concentrations available; ii) only cell or tissue studies present; iii) non-human studies; iv) control condition considered as not healthy (e.g. pregnancy or patients with another condition); v) patients below the age of 18 years; or vi) only mean IL-38 concentrations stated (no range or SD/SEM presented).

### Data extraction

When a publication fulfilled the inclusion criteria, the following data were obtained: i) mean blood concentrations of IL-38 concentration in pg/mL with the accessory standard deviation (SD), standard error of the mean (SEM) or data range; ii) type of collection tube (serum or EDTA, or heparin plasma); iii) number of healthy subjects; and if available: iv) sex and age distributions, and v) the ethnicity of the subjects or the country where the study was performed. Lastly, also the manufacturer of the ELISA kits was noted.

### Ethics

Inclusion of healthy subjects was approved by the local institutional review board (CMO region Arnhem-Nijmegen, #2299 2010/104) and conducted according to the principles of the International Conference on Harmonization-Good Clinical Practice guidelines and the declaration of Helsinki.

### Additional cohorts

In order to generate more reference cohorts, previously stored plasma from existing cohorts of healthy volunteers was used. Some of these cohorts were intervention studies, but in this case baseline samples before the intervention were used to determine IL-38 in. These cohorts are numbered from 1 to 7 and are presented in **Table 1**. For cohort 6 and 7 see also: www.humanfunctionalgenomics.org.

**Table 1:** An overview of the characteristics of the validation cohorts in which IL-38 concentrations were measured.

	N	Sex	Age	BMI
Cohort 1 (14)	21	Both	20-36	N/A
Cohort 2 (15)	30	Male	19-37	N/A
Cohort 3 (16)	74	Female	18-48	N/A
Cohort 4, “300BCG study” (17)	320	183 Female	18-75	N/A
Cohort 5 [this study]	204	151 Female	38-88	Yes [18.7-46.1]
Cohort 6, “200FG study” (5, 17)	225	54 Female	23-73	Yes [19.6-37.2]
Cohort 7, “500FG study” (18)	288	167 Female	18-75	N/A

N/A, not available.

### Blood collection

In order to compare the effects of different collection tubes, the effect of hemolysis, and the effect of freeze thawing on the detectability of IL-38, fresh blood was collected from healthy volunteers, into EDTA, heparin, and serum tubes. To induce hemolysis, 1 to 2 mL of blood was collected in a 10ml-EDTA tube, where the vacuum of the tube induced red cell lysis. 10 minutes after collection, blood was spun at 2900 g for 10 minutes at room temperature. Serum tubes were left at room temperature for 45 minutes after blood collection before centrifugation. Supernatant was collected and freeze-thawed for 0 to 3 times from room temperature to -20°C.

Hemolysis was confirmed by determining the LDH level in the samples by an LDH-cytotoxicity assay kit (MAK380, Sigma Aldrich).

IL-38 concentrations were determined by the human IL-38/IL1F10 DuoSet ELISA (R&D Systems, Inc., Minneapolis, MN, USA) according to the manufacturer’s instructions with minor adaptations. Briefly, an extended standard curve of 7.8 - 2000 pg/mL was used, yielding 15.6 pg/mL as lower and 4000 pg/mL as upper limit of quantification due to the sample dilution step. Samples were diluted 1:1 in PBS containing 1% BSA (Sigma-Aldrich, Germany), and samples and standard were incubated overnight at 4°C on pre-coated 96-well plates. Values below detection limit were included in the analyses using the lower limit of quantification as assigned value.

### eQTL analyses

Significant expression qualitative trait loci (eQTLs) in *IL1F10* were identified using different population-based variant databases, namely GTEx, gnomAD (19), and an “inhouse” database consisting of >30,000 clinical exons run at the diagnostics division of the Human Genetics department of the Radboud University Medical Center (Radboudumc). First, GTEx was sourced for significant eQTLs in *IL1F10* (Significant Single-Tissue eQTLs for *IL1F10* (ENSG00000136697.12) in all tissues Data Source: GTEx Analysis Release V8 (dbGaP Accession phs000424.v8.p2 on 04.04.2022)), resulting in 392 hits (**Supplementary Table 1**). To match identified variants to allele frequencies in different ethnicities, the variant set was annotated using our inhouse annotator, resulting in only 12 observed variants in the GRCh38 build. From 12 significant eQTLs with available frequency data, two were located in the exon region of *IL1F10* and characterized as missense mutations (**Supplementary Table 2**). Identified SNPs were validated in gnomAD. To assess whether either of the variants affects circulating IL-38 plasma concentrations, the genotypes were matched to IL-38 concentrations measured by ELISA in the 200FG cohort (**Supplementary Figure 1**).

### Statistical analysis

All data were collected in a Microsoft Excel spread sheet (version 16.57). To determine a mean value from the IL-38 concentrations in the healthy adults from the included studies, the mean of each of these studies was weighted by its corresponding sample size. By dividing the sum of these weighted means by the total sample size a weighted mean was calculated.

In order to determine the corresponding lower and upper limits with this weighted mean of IL-38 concentrations, we first calculated these limits for each individual study. When no upper and lower limits were presented, they were calculated by mean ± 2 SD. SEM was converted to SD by multiplying the SEM by the square root of the corresponding sample number. To calculate a weighted upper and lower limit the same formula was applied as to calculate the weighted mean.

Data were presented in GraphPad Prism version 6 (GraphPad Software, San Diego, CA, USA). In order to define and calculate outliers, the applicable data are displayed in a box plot with whiskers and outliers were calculated by using the interquartile Tukey fence method. Differences between two groups were calculated by using Wilcoxon matched-pairs signed rank test. Correlation calculations were performed by linear regression with a 95% confidence interval shown.

## Results

### Overview of data from literature

**Figure 1:**
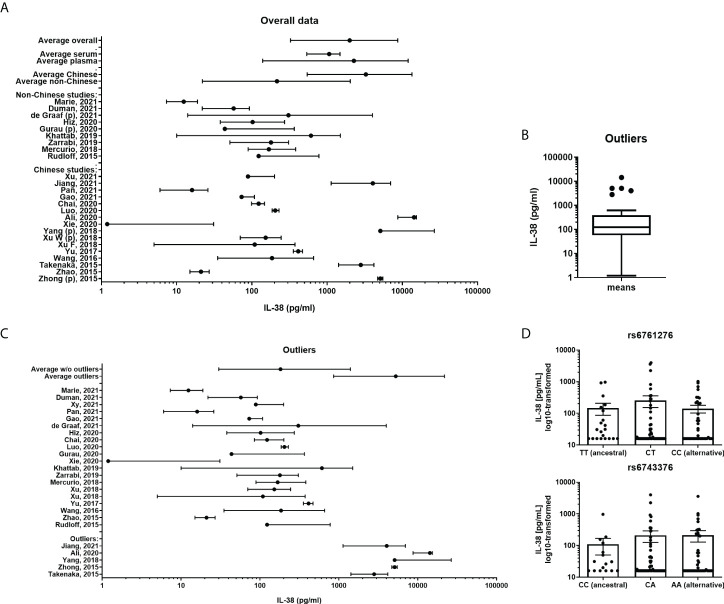
IL-38 concentrations reported for healthy cohorts. **(A)** A presentation of all included cohorts. IL-38 data are presented as mean and range. A weighed average was calculated. Subgroup analyses were made divided by serum or plasma (marked “p”) concentrations of IL-38 and by geographical location. **(B)** Outliers of all included cohorts were calculated by using the interquartile Tukey fence method. **(C)** A presentation of all included cohorts without the defined outliers and the outliers separately, IL-38 data are presented as mean and range. A weighted average was calculated. **(D)** IL-38 plasma concentrations stratified by haplotype of rs6743376 and rs6761276.

Between September 2015 and December 2021 25 published studies (**Table 2**) fulfilled the inclusion criteria as described in the Methods section (20–44). In order to determine the distribution of the collected data an overview figure was made (**Figure 1A**). In this panel, it can already be observed that there are major differences between several studies concerning the distribution of the cytokine concentrations. Comparing the two studies for which extreme differences were reported (Xie et al. and Ali et al.), mean IL-38 concentrations differed over 10.000-fold (31, 33). We next considered potential factors that may cause these differences. Firstly, we determined whether overall IL-38 concentrations differed between studies that use either serum or plasma samples. Most studies were performed using serum samples (80%, **Table 2**); however, no major differences were observed between IL-38 in plasma and serum samples (**Figure 1A**). Secondly, we determined whether the genetic background of the healthy subjects could explain the observed differences. As most of the studies were performed in China, we divided the included studies in Chinese and non-Chinese (**Figure 1A**) which showed a difference between the two groups. However, this trend seemed mostly to rely on a few outlier studies that were present in the Chinese group. These outliers could not be contributed to the use of an ELISA kit by a certain manufacturer, or to the distribution of sex amongst the cohorts (**Table 1**; **Supplementary Figure 1**). Next, the outliers were statistically defined by using the interquartile Tukey fence method (**Figure 1B**). When presenting the data without these outliers, a more homogenous pattern was observed (**Figure 1C**). Still, differences of 10-fold up to 100-fold were observed between the means in these studies. This therefore raises the question whether these data are sufficiently reliable to build IL-38 reference values on.

**Table 2:** An overview of the study and healthy volunteer characteristics of all included studies.

Study	Plasma/Serum^1^	ELISA	Nationality	Detectable (%)	Mean (pg/mL)	N	Male	Age
Rudloff, 2015	Serum	Adipogen	Australian	39	123	28	?	?
Zhong, 2015	Plasma (heparin)	Adipogen	Chinese		5100	26	19	55 (SD: 6,5)
Zhao, 2015	Serum	Ray Biotech	Chinese		21	9	5	66 (range: 55-78)
Takenaka, 2015	Serum	Self-made	Chinese	9	2800	56	33	?
Wang, 2016	Serum	Cusabio life sciences	Chinese		185	43	34	34 (range: 22-40)
Yu, 2017	Serum	Cusabio life sciences	Chinese		413	23	0	29 (SD: 0,85)
Xu W, 2018	Plasma (?)	Adipogen	Chinese		152	53	6	52 (range: 39-63)
Yang, 2018	Plasma (?)	Adipogen	Chinese		5100	408	408	57 (range: 51-65)
Mercurio, 2018	Serum	BD Pharmingen	Italian		168	25	?	18-70
Xu F, 2018	Serum	R&D Systems	Chinese		109	29	18	46 (range: 33-57)
Khattab, 2019	Serum	Adipogen	Egypt		610	26	13	47 (range: 12-65)
Zarrabi, 2019	Serum	R&D Systems	Iran		179	81	24	40
Ali, 2020	Serum	Eastpharm	Chinese		14290	27	15	?
Xie, 2020	Serum	R&D Systems	Chinese	5	1,19	43	20	33 (range: 22-64)
Luo, 2020	Serum	R&D Systems	Chinese		204	13	3	43 (range: 31-55)
Chai, 2020	Serum	R&D Systems	Chinese		123	11	6	41 (SD: 36)
Hiz, 2020	Serum	Sunred bio	Turkey		102	40	27	55 (range: 23-79)
Gurau, 2020	Plasma (?)	Wuhan Fine biotech co	Italian		43,6	65	31	65 (SD: 9,5)
Gao, 2021	Serum	?	Chinese		73	59	?	?
de Graaf, 2021	Plasma (EDTA)	Biotechne	Dutch	67	306	288	119	23 (OQR 21-27)
Jiang, 2021	Serum	Phoenix Peptides	Chinese		4050	25	15	59 (SD: 7)
Pan, 2021	Serum	R&D Systems	Chinese		16	12	7	27 (range: 22-42)
Xu, 2021	Serum	Shanghai Jianglai Biotech Co	Chinese		88,7	50	34	36,4 (SD: 11,8)
Duman, 2021	Serum	Sunred bio	Turkey		57,2	60	0	50-67
Marie, 2021	Serum	Sunred bio	Egypt		12,4	21	6	12-50

^1^Questionmarks refer to studies with unspecified anticoagulant.

Other potentially confounding factors such as sex, BMI, and age were considered too, but unfortunately most data were not sufficiently presented to also take these factors along (**Table 2**).

### eQTL analysis

In our comparison it stood out that markedly higher IL-38 concentrations were reported in studies performed in Chinese populations, indicating that the genetic background could be a confounding factor. Assessing publicly available databases for eQTLs, we identified two significant variants in the coding region of *IL1F10*, one of which with higher allele frequency in non-Finnish Europeans than in East Asian, but not South Asian populations (**Supplementary Table 2**). However, matching IL-38 concentrations to the haplotypes of both variants showed that protein concentrations are comparable between carriers of ancestral (rs6761276 – TT; rs6743376 – CC) and alternative alleles (rs6761276 – TC, CC; rs6743376 – CA, AA) (**Figure 1D**). Thus, ethnicity is unlikely to explain the big differences in circulating IL-38 observed between the cohorts included in our analyses.

### Blood collection conditions

**Figure 2:**
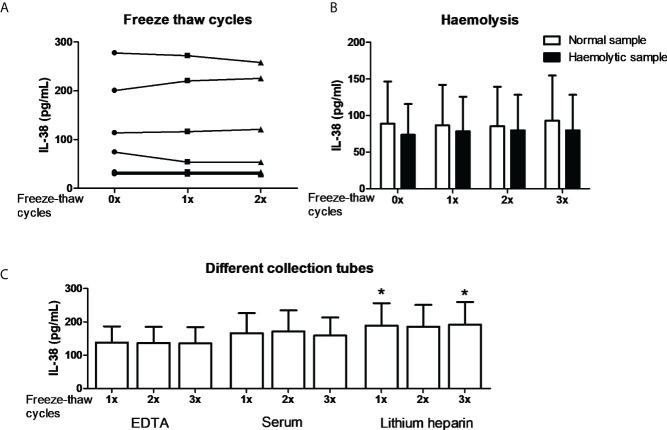
IL-38 concentrations and the effect of blood collection conditions. **(A)** IL-38 concentrations in EDTA serum were determined directly or after one or two freeze-thaw cycles between -80 degrees Celsius and room temperature. N=8. **(B)** Haemolytic plasma was compared to the corresponding non-haemolytic plasma, directly or after up to three freeze-thaw cycles. N=4. **(C)** Blood was collected in EDTA, serum, and heparin tubes and IL-38 concentrations were determined directly or after up to three freeze-thaw cycles. N=12, Wilcoxon matched-pairs signed rank test, *p<0,05 compared to the corresponding sample of both EDTA and serum tubes.

In order to further substantiate the collection conditions as confounding factors we performed exploratory experiments. Firstly, it is well established that certain cytokines degrade by repeated freeze-thawing (45, 46). We therefore collected blood in EDTA tubes, and plasma was either subjected to one or two freeze-thaw cycles to -20 degrees or directly used for IL-38 determination by ELISA. No major differences were found among different subjects which shows that IL-38 is relatively stable among freeze-thaw cycles (**Figure 2A**). Next, we determined the potential role of hemolysis on the assay (47). One to two mL of blood was collected in a 10 mL-EDTA collection tube; hence the vacuum would induce hemolysis, which was confirmed by an LDH assay (data not shown). Additionally, the plasma was subjected to up to three freeze-thaw cycles. The data shows that neither hemolysis nor the freeze-thaw cycles influenced IL-38 concentrations (**Figure 2B**). Lastly, different anti-coagulants in blood collection tubes can be a confounding factor (46). Blood from the same healthy volunteers was collected in EDTA, heparin, or serum tubes, and plasma or serum was subjected to up to three freeze-thaw cycles. Again, the number of freeze-thaw cycles did not affect the concentrations of IL-38 (**Figure 2C**). We did, however, find significantly more IL-38 in plasma collected in heparin tubes compared to EDTA and serum tubes (188.9 pg/mL ± 67.1 versus 137.8 pg/mL ± 48.6 and 166.2 pg/mL ± 59.9, respectively; mean ± SEM). However, given this small overall difference this is again unlikely to cause the differences observed in **Figure 1**.

### Additional cohorts

In the last decade we have collected blood samples from several different cohorts in our laboratory of which more detailed healthy volunteer characteristics are available. In order to determine whether other potential confounding factors namely BMI, age, and sex could influence IL-38 concentrations, this information was collected and IL-38 concentrations from EDTA plasma were measured in these cohorts using R&D systems DuoSet ELISA kits.

**Figure 3:**
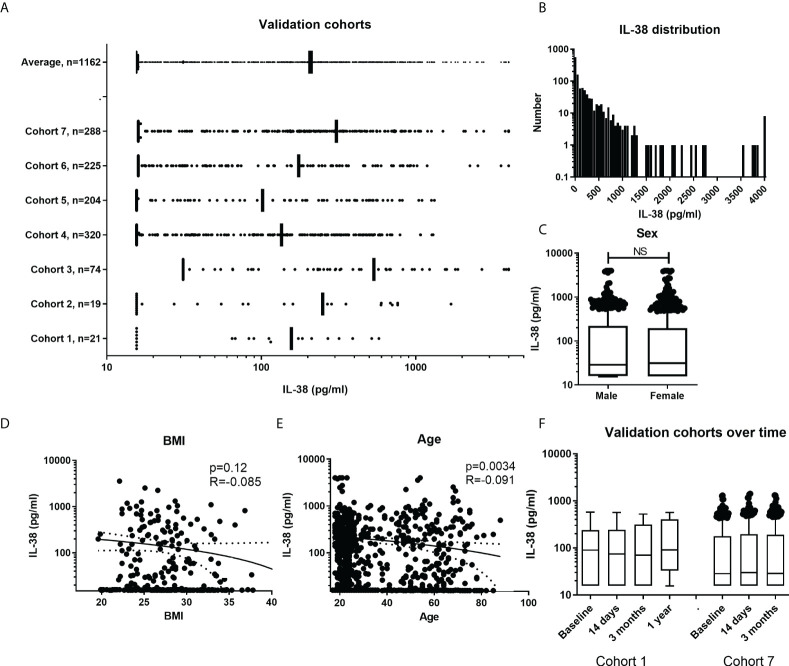
IL-38 concentrations in additional cohorts. **(A)** Overall presentation of the individual data point in the different cohorts. Data presented as IQRs and outliers defined by using the interquartile Tukey fence method. **(B)** Frequency distribution of IL-38 concentrations with a bin width of 50pg/ml. **(C)** IL-38 data divided by sex. Unpaired t-test. **(D, E)** Distribution of IL-38 concentrations by BMI (N=325) and age (N=1024). Linear regression displayed with a 95% confidence band. **(F)** Presentation of two cohorts with multiple time points. Data presented as IQRs and outliers defined by using the interquartile Tukey fence method, Wilcoxon matched-pairs signed rank test. NS, Not Statistically Significant.

Firstly, an overview figure was made with the different cohorts and their distribution of the individual data points, n=1162 (**Figure 3A**). It can be observed that the distribution is quite homogenous among the cohorts. The overall distribution is in the lower ranges compared to the data from cohorts presented in **Figure 1C**. As individual data points were available in these cohorts, the distribution could be determined. **Figure 3B** shows the distribution of the IL-38 concentrations against the standard curve used in these experiments (detection range: 15.6 to 4000 pg/mL; detection range cohort 3: 31.2 to 4000 pg/mL). Decreasing the lower limit could potentially increase the determination of the data distribution. We show that sex does not influence IL-38 concentrations in these cohorts (**Figure 3C**). BMI shows a trend towards higher BMI corresponding with lower IL-38 concentrations (**Figure 3D**), as was observed before (28). Also, age shows an inverse correlation with IL-38 concentrations (**Figure 3E**), which also corresponds with previously published data (28, 43). Lastly, we determined whether IL-38 remains stable over time in two cohorts for which multiple consecutive samples were available. The data show no significant differences amongst the sequential timepoints (**Figure 3F**).

## Discussion

IL-38 is the latest added member of the IL-1 family of cytokines, but in the last few years it has already been shown to be dysregulated in a wide range of diseases such as cancer, infectious diseases, and autoimmunity (6, 7). Patients with atopic dermatitis (20), influenza (21), SLE (22), rheumatoid arthritis (25, 39), myocardial infarction (23), sepsis (24), acquired immune-mediated neuropathies (31), osteoarthritis (32), acute respiratory distress syndrome (35), inflammatory bowel disease (33), type 2 diabetes (41), and vitiligo (43) present with higher blood concentrations of IL-38. This in contrast to e.g., patients with Graves’ and Hashimoto’s thyroiditis (44), Sjögren’s disease (34), Behçet’s disease (37), chronic brucellosis (40), and psoriasis (27), in whom IL-38 circulating concentrations were lower compared to healthy volunteers.

Also, the course of IL-38 appears to be relevant in certain diseases. Continuously high IL-38 concentrations are correlated with complex SLE disease with more organ (e.g. kidney and cerebral) complications (22). In diseases in which patients present with high baseline IL-38, a reduction of IL-38 after treatment is a predictor for treatment response in e.g., reperfusion therapy in myocardial infarction (23), in treatment of rheumatoid arthritis (25), and in juvenile SLE patients treatment with prednisone reduced IL-38 concentrations (48). In contrast, in psoriasis, in which patients present with reduced IL-38, the upregulation of IL-38 was correlated with treatment response (27). In atherosclerotic disease, IL-38 concentrations were also higher compared to healthy volunteers, and higher concentrations of IL-38 predicted better response to statin therapy in these patients (26). A comparable observation was made in hepatitis B patients treated with telbivudine (29).

In most of these studies, IL-38 was used as a biomarker for disease activity and potential therapy response. However, given the anti-inflammatory properties of IL-38, treatment with IL-38 supplementation was hypothesized in many of these reports. In the context of lower IL-38 concentrations in patients with the disease, this is a logical hypothesis and was shown effective in e.g., peripheral blood mononuclear cells (PBMCs) from patients with Sjögren’s syndrome (34) and patients with hyperlipidemia (26). Interestingly, also in patients with already high concentrations of IL-38, additional supplementation is hypothesized to be beneficial by increasing the anti-inflammatory signal even further. In this respect, administration of IL-38 in a murine sepsis model resulted in increased survival (24), and over-expression of IL-38 in a murine osteoarthritis model inhibited the disease activity (32). Moreover, in an *in vivo* murine model of inflammatory bowel disease, treatment with recombinant IL-38 reduced important inflammatory mediators of the disease from colonic samples (33).

We have not been able to find any information in the literature that define normal reference values for IL-38. Several groups have, however, tried to define cut-offs for the diagnosis of certain diseases or treatment responses. A cut-off value of 342 pg/mL was defined for the diagnosis of rheumatoid arthritis, with a sensitivity and specificity, respectively of 72% and 91% (25). In a cohort of chronic hepatitis B patients, high concentrations of serum IL-38 (>250 pg/ml) at baseline were associated with a greater probability of viral response to treatment at 24 weeks (48.15% vs 15.79%) (29). However, given the major differences in IL-38 concentrations between the different studies cited here, the question remains how useful these cut-offs will be in the general population.

Apart from the disease, other potential confounding factors should be considered while determining a reference range for IL-38. Firstly, we assessed whether *IL1F10* expression could differ between different ethnicities using publicly available eQTL databases. Only one significant variant in the coding region of *IL1F10* presented with higher allele frequency in individuals with East Asian ancestry compared to non-Finnish Europeans. Thus, ethnicity is unlikely to explain the big differences observed between the cohorts included in our analyses. In our additional experiments we have tried to define or rule out common influencing factors. We have shown that freeze-thawing, hemolysis, and collection of serum or plasma has no major effect on IL-38 concentrations. We did find, however, higher concentrations of IL-38 when blood was collected in heparin tubes compared to EDTA or serum tubes, but although statistically different, the absolute differences were only minor and could not explain the major differences found between the several cited studies. We did not find differences in IL-38 concentrations between males and females, just as others have shown before (28, 31). Age showed a negative correlation with IL-38 concentrations, and in BMI a (although not significant) negative trend was observed. These observations were also comparable with prior studies (28, 41, 43). Lastly, no major variation over longer time courses was observed, which had also been shown before in 48 subjects over a time period of a year (28).

Given the major differences that we have observed between the included studies, and the exclusion of common relevant confounding factors, it is essential to determine potential methodological errors as well as the impact of ELISA kit types and manufacturers. Non-specific binding is for example a relevant factor that could interfere with assay specificity (49). For example, we previously reported that the IL-38 ELISA used by our laboratory detects 55% of a known concentration of recombinant IL-38 which we attributed to plasma-factors binding to IL-38 (28). Interestingly, there appears to be a great difference in the detectability of IL-38, with a variation from 5% (33, 48), to 9% (39), to 49% (22), to 67% (28), to probably 100% in the other studies. Also, in our validation cohorts we found a detectability of 56% with a lower cut off at 15.6 pg/mL. The distribution curve presented in **Figure 3B** shows that most subjects have IL-38 concentrations around the lower limit of detection. It is therefore imaginable that studies reporting very low mean concentrations (33, 43) might report background noise as IL-38 values. This suggests that a more sensitive ELISA that could determine even lower concentrations of IL-38 could generate a more reliable image of the distribution of IL-38 in healthy adults. As for the type of ELISA kit used, we could not group identified outlier studies as well as studies with outstandingly high or low IL-38 concentrations based on the used ELISA kit manufacturer. Noteworthy, information concerning the type of detection or coating antibodies used in the different ELISA kits, which may display different binding affinities to cleaved IL-38, was not available in all cases and can therefore not be ruled out as confounding factor.

Based on the available data, it was not possible to define a reliable refence range for IL-38 in healthy volunteers, as was possible for the IL-37 data extracted from literature (13). This is remarkable as IL-37 has several isoforms that will probably not be determined by all ELISA kits (50, 51). However, to give an estimation of a potential direction of a reference range of IL-38 we could rely on the data generated after exclusion of outliers. The reference values extracted from literature lead to a range from 30.0 to 1403.2 pg/mL. The data from the validation experiments performed in our lab result in a 5^th^ and 95^th^ percentile of 15.6 and 819.9 pg/mL (range from 16.6 to 2673.5 pg/mL), where it should be noted that 15.6 pg/mL is the lower detection limit of the ELISA kit. Only considering studies using the most common ELISA kit from R&D Systems, a reference range from 20.0 to 2328.2 pg/mL can be set. It has to be noted though, that 54% of these are studies performed in our institute.

In conclusion, we have performed an analysis of all available studies reporting IL-38 concentrations of healthy subjects in literature in order to define a reference range in healthy volunteers. However, given the relatively large differences between several studies we have not been able to define such a range yet. While using ELISA to determine cytokine levels, it is essential to verify the specificity of the method to confirm the reliability of the technique. This could be achieved by using a reference sample with a known concentration or by performing a spike experiment to generate a percentage of recovery for a certain experiment.

## Data availability statement

The original contributions presented in the study are included in the article/**Supplementary Materials**. Further inquiries can be directed to the corresponding author.

## Ethics statement

The studies involving human participants were reviewed and approved by CMO region Arnhem-Nijmegen, #2299 2010/104. The patients/participants provided their written informed consent to participate in this study.

## Author contributions

All authors directly made a substantial and intellectual contribution to the work. DG, LT, and RA contributed to conception and design of the study. DG and RA performed the data extraction, and LT and RA performed the statistical analysis. LT performed the laboratory experiments and genetic analyses. RA wrote the first draft, and LT wrote sections of the manuscript. All authors contributed to manuscript revision.

## Funding

D.M.G. is supported by the Interleukin foundation. R.J.A. is supported by the VENI grant (09150161810007). C.A.D. is supported by NIH grant AI-15614. M.G.N. is supported by the European Research Council (ERC, #833247) and Spinoza Price of the Dutch Research Council (NWO SPI 94- 212). L.A.B.J. is supported by a Competitiveness Operation Program grant of the Romanian Ministry of European Funds (HINT, ID P_37_762; MySMIS 103587).

## Conflict of interest

The authors declare that the research was conducted in the absence of any commercial or financial relationships that could be construed as a potential conflict of interest.

## Publisher’s note

All claims expressed in this article are solely those of the authors and do not necessarily represent those of their affiliated organizations, or those of the publisher, the editors and the reviewers. Any product that may be evaluated in this article, or claim that may be made by its manufacturer, is not guaranteed or endorsed by the publisher.

## References

[1] Bensen JT Dawson PA Mychaleckyj JC Bowden DW. Identification of a novel human cytokine gene in the interleukin gene cluster on chromosome 2q12-14. J Interferon Cytokine Res (2001) 21:899–904. doi: 10.1089/107999001753289505 11747621

[2] van de Veerdonk FL Stoeckman AK Wu G Boeckermann AN Azam T Netea MG. IL-38 binds to the IL-36 receptor and has biological effects on immune cells similar to IL-36 receptor antagonist. Proc Natl Acad Sci USA (2012) 109:3001–5. 10.1073/pnas.1121534109 PMC3286950 22315422

[3] Mora J Schlemmer A Wittig I Richter F Putyrski M Frank AC. Interleukin-38 is released from apoptotic cells to limit inflammatory macrophage responses. J Mol Cell Biol (2016) 8:426–38. doi: 10.1093/jmcb/mjw006 26892022

[4] de Graaf DM Maas RJA Smeekens SP Eisenmesser E Redzic JS Helsen MM. Human recombinant interleukin-38 suppresses inflammation in mouse models of local and systemic disease. Cytokine (2021) 137:155334. doi: 10.1016/j.cyto.2020.155334 33128926 PMC7725974

[5] de Graaf DM Teufel LU van de Veerdonk FL Joosten LAB Netea MG Dinarello CA. IL-38 prevents induction of trained immunity by inhibition of mTOR signaling. J Leukoc Biol (2021) 110:907–15. doi: 10.1002/JLB.3A0220-143RRR PMC8380748 33620105

[6] de Graaf DM Teufel LU Joosten LAB Dinarello CA. Interleukin-38 in health and disease. Cytokine (2022) 152:155824. doi: 10.1016/j.cyto.2022.155824 35220115

[7] Diaz-Barreiro A Huard A Palmer G. Multifaceted roles of IL-38 in inflammation and cancer. Cytokine (2022) 151:155808. doi: 10.1016/j.cyto.2022.155808 35066449

[8] Monastero RN Pentyala S. Cytokines as biomarkers and their respective clinical cutoff levels. Int J Inflam (2017) 2017:4309485. doi: 10.1155/2017/4309485 28487810 PMC5401738

[9] Kim HO Kim HS Youn JC Shin EC Park S. Serum cytokine profiles in healthy young and elderly population assessed using multiplexed bead-based immunoassays. J Transl Med (2011) 9:113. doi: 10.1186/1479-5876-9-113 21774806 PMC3146842

[10] Li Y Yi JS Russo MA Rosa-Bray M Weinhold KJ Guptill JT. Normative dataset for plasma cytokines in healthy human adults. Data Brief (2021) 35:106857. doi: 10.1016/j.dib.2021.106857 33665253 PMC7900339

[11] Italiani P Puxeddu I Napoletano S Scala E Melillo D Manocchio S. Circulating levels of IL-1 family cytokines and receptors in alzheimer's disease: new markers of disease progression? J Neuroinflamm (2018) 15:342. doi: 10.1186/s12974-018-1376-1 PMC6292179 30541566

[12] Kleiner G Marcuzzi A Zanin V Monasta L Zauli G. Cytokine levels in the serum of healthy subjects. Mediators Inflamm (2013) 2013:434010. doi: 10.1155/2013/434010 23533306 PMC3606775

[13] Santarelli DM Vincent FB Rudloff I Nold-Petry CA Nold MF Russo MA. Circulating interleukin-37 levels in healthy adult humans - establishing a reference range. Front Immunol (2021) 12:708425. doi: 10.3389/fimmu.2021.708425 34367169 PMC8343013

[14] Kleinnijenhuis J Quintin J Preijers F Joosten LA Ifrim DC Saeed S. Bacille calmette-guerin induces NOD2-dependent nonspecific protection from reinfection *via* epigenetic reprogramming of monocytes. Proc Natl Acad Sci U S A (2012) 109:17537–42. 10.1073/pnas.1202870109 PMC3491454 22988082

[15] Arts RJW Moorlag SJCF Novakovic B Li Y Wang SY Oosting M. BCG Vaccination protects against experimental viral infection in humans through the induction of cytokines associated with trained immunity. Cell Host Microbe (2018) 23:89–100.e5. doi: 10.1016/j.chom.2017.12.010 29324233

[16] Blok BA de Bree LCJ Diavatopoulos DA Langereis JD Joosten LAB Aaby P. Interacting, nonspecific, immunological effects of bacille calmette-guérin and tetanus-diphtheria-pertussis inactivated polio vaccinations: An explorative, randomized trial. Clin Infect Dis (2020) 70:455–63. 10.1093/cid/ciz246 30919883

[17] Moorlag SJCF Matzaraki V van Puffelen JH van der Heijden C Keating S Groh L. An integrative genomics approach identifies KDM4 as a modulator of trained immunity. Eur J Immunol (2021) 52:431–46. doi: 10.1002/eji.202149577 34821391 PMC9299854

[18] Li Y Oosting M Smeekens SP Jaeger M Aguirre-Gamboa R Le KTT. A functional genomics approach to understand variation in cytokine production in humans. Cell (2016) 167:1099–110.e14. doi: 10.1016/j.cell.2016.10.017 27814507

[19] Karczewski KJ Francioli LC Tiao G Cummings BB Alföldi J Wang Q. The mutational constraint spectrum quantified from variation in 141,456 humans. Nature (2020) 581:434–43. doi: 10.1038/s41586-020-2308-7 PMC7334197 32461654

[20] Khattab F Khater E Ibraheem H. Serum levels of interleukin-38 in sufferers with atopic eczema. Egyptian J Dermatol Venerology (2019) 2(39):66–70. doi: 10.4103/ejdv.ejdv_2_18

[21] Gao X Chan PKS Lui GCY Hui DSC Chu IM Sun X. Interleukin-38 ameliorates poly(I:C) induced lung inflammation: therapeutic implications in respiratory viral infections. Cell Death Dis (2021) 12:53. doi: 10.1038/s41419-020-03283-2 33414457 PMC7790341

[22] Rudloff I Godsell J Nold-Petry CA Harris J Hoi A Morand EF. Brief report: Interleukin-38 exerts antiinflammatory functions and is associated with disease activity in systemic lupus erythematosus. Arthritis Rheumatol (2015) 67:3219–25. doi: 10.1002/art.39328 26314375

[23] Zhong Y Yu K Wang X Ji Q Zeng Q. Elevated plasma IL-38 concentrations in patients with acute ST-segment elevation myocardial infarction and their dynamics after reperfusion treatment. Mediators Inflammation (2015) 2015:490120. doi: 10.1155/2015/490120 PMC4706979 26819499

[24] Xu F Lin S Yan X Wang C Tu H Yin Y. Interleukin 38 protects against lethal sepsis. J Infect Dis (2018) 218:1175–84. doi: 10.1093/infdis/jiy289 29762676

[25] Xu WD Su LC He CS Huang AF. Plasma interleukin-38 in patients with rheumatoid arthritis. Int Immunopharmacol (2018) 65:1–7. doi: 10.1016/j.intimp.2018.09.028 30268016

[26] Yang N Song Y Dong B Li Y Kou L Yang J. Elevated interleukin-38 level associates with clinical response to atorvastatin in patients with hyperlipidemia. Cell Physiol Biochem (2018) 49:653–61. doi: 10.1159/000493029 30165364

[27] Mercurio L Morelli M Scarponi C Eisenmesser EZ Doti N Pagnanelli G. IL-38 has an anti-inflammatory action in psoriasis and its expression correlates with disease severity and therapeutic response to anti-IL-17A treatment. Cell Death Dis (2018) 9:1104. doi: 10.1038/s41419-018-1143-3 30377293 PMC6207563

[28] de Graaf DM Jaeger M van den Munckhof ICL Ter Horst R Schraa K Zwaag J. Reduced concentrations of the b cell cytokine interleukin 38 are associated with cardiovascular disease risk in overweight subjects. Eur J Immunol (2021) 51:662–71. doi: 10.1002/eji.201948390 PMC7983920 33125159

[29] Wang HJ Jiang YF Wang XR Zhang ML Gao PJ. Elevated serum interleukin-38 level at baseline predicts virological response in telbivudine-treated patients with chronic hepatitis b. World J Gastroenterol (2016) 22:4529–37. doi: 10.3748/wjg.v22.i18.4529 PMC4858634 27182162

[30] Yu Z Liu J Zhang R Huang X Sun T Wu Y. IL-37 and 38 signalling in gestational diabetes. J Reprod Immunol (2017) 124:8–14. doi: 10.1016/j.jri.2017.09.011 28992508

[31] Ali ZPM Ghafouri-Fard S Komaki A Mazdeh M Taheri M Eftekharian MM. Assessment of IL-38 levels in patients with acquired immune-mediated polyneuropathies. J Mol Neurosci (2020) 70:1385–8. doi: 10.1007/s12031-020-01558-z 32367504

[32] Jiang L Zhou X Huang C Bao J Li J Xu K. The elevated expression of IL-38 serves as an anti-inflammatory factor in osteoarthritis and its protective effect in osteoarthritic chondrocytes. Int Immunopharmacol (2021) 94:107489. doi: 10.1016/j.intimp.2021.107489 33774357

[33] Xie C Yan W Quan R Chen C Tu L Hou X. Interleukin-38 is elevated in inflammatory bowel diseases and suppresses intestinal inflammation. Cytokine (2020) 127:154963. doi: 10.1016/j.cyto.2019.154963 31927461

[34] Luo D Chen Y Zhou N Li T Wang H. Blockade of Th17 response by IL-38 in primary sjögren's syndrome. Mol Immunol (2020) 127:107–11. doi: 10.1016/j.molimm.2020.09.006 32950755

[35] Chai YS Lin SH Zhang M Deng L Chen Y Xie K. IL-38 is a biomarker for acute respiratory distress syndrome in humans and down-regulates Th17 differentiation *in vivo* . Clin Immunol (2020) 210:108315. doi: 10.1016/j.clim.2019.108315 31756565

[36] Pan Y Wang M Chen X Chen Y Ai S Su W. Elevated IL-38 inhibits IL-23R expression and IL-17A production in thyroid-associated ophthalmopathy. Int Immunopharmacol (2021) 91:107300. doi: 10.1016/j.intimp.2020.107300 33383445

[37] Zarrabi M Gholijani N Shenavandeh S Aflaki E Amirghofran Z. IL-38 serum levels in patients with behcet's disease and the relationship with clinical features. Eur Cytokine Netw (2019) 30:82–7. 10.1684/ecn.2019.0430 31957702

[38] Zhao B Chen W Jiang R Zhang R Wang Y Wang L. Expression profile of IL-1 family cytokines in aqueous humor and sera of patients with HLA-B27 associated anterior uveitis and idiopathic anterior uveitis. Exp Eye Res (2015) 138:80–6. doi: 10.1016/j.exer.2015.06.018 26116905

[39] Takenaka SI Kaieda S Kawayama T Matsuoka M Kaku Y Kinoshita T. IL-38: A new factor in rheumatoid arthritis. Biochem Biophys Rep (2015) 4:386–91. doi: 10.1016/j.bbrep.2015.10.015 PMC5669445 29124228

[40] Hiz P Kanbur E Demir N Akalin H Cagan E Pashazadeh M. Roles of novel IL-1 family (IL-36, IL-37, and IL-38) members in chronic brucellosis. Cytokine (2020) 135:155211. doi: 10.1016/j.cyto.2020.155211 32736334

[41] Gurău F Silvestrini A Matacchione G Fazioli F Bonfigli AR Olivieri F. Plasma levels of interleukin-38 in healthy aging and in type 2 diabetes. Diabetes Res Clin Pract (2021) 171:108585. doi: 10.1016/j.diabres.2020.108585 33310128

[42] Duman BA Duman S Çamurcu Y Gem M Erdinç L. Evaluation of serum interleukin-38 levels in different radiographic grades of idiopathic knee osteoarthritis. J Interferon Cytokine Res (2021) 41:425–30. doi: 10.1089/jir.2020.0109 34788133

[43] Mahmoud Marie RE Adel AM Abd El-Fadeal NM Eyada MMK. Interleukin 38 serum level is increased in patients with vitiligo, correlated with disease severity, and associated with signs of disease activity. J Cosmet Dermatol (2021) 15. doi: 10.1111/jocd.14612 34783147

[44] Xu J Huang G Weng L Gong L Mao Y Li Y. Low serum interleukin-38 levels in patients with graves' disease and hashimoto's thyroiditis. J Clin Lab Anal (2022) 36:e24101. doi: 10.1002/jcla.24101 34799942 PMC8761401

[45] Simpson S Kaislasuo J Guller S Pal L. Thermal stability of cytokines: A review. Cytokine (2020) 125:154829. doi: 10.1016/j.cyto.2019.154829 31472404

[46] de Jager W Bourcier K Rijkers GT Prakken BJ Seyfert-Margolis V. Prerequisites for cytokine measurements in clinical trials with multiplex immunoassays. BMC Immunol (2009) 10:52. doi: 10.1186/1471-2172-10-52 19785746 PMC2761376

[47] Karsten E Breen E Herbert BR. Red blood cells are dynamic reservoirs of cytokines. Sci Rep (2018) 8:3101. doi: 10.1038/s41598-018-21387-w 29449599 PMC5814557

[48] Takeuchi Y Seki T Kobayashi N Sano K Shigemura T Shimojo H. Analysis of serum IL-38 in juvenile-onset systemic lupus erythematosus. Mod Rheumatol (2018) 28:1069–72. doi: 10.1080/14397595.2018.1436118 29385862

[49] Güven E Duus K Lydolph MC Jørgensen CS Laursen I Houen G. Non-specific binding in solid phase immunoassays for autoantibodies correlates with inflammation markers. J Immunol Methods (2014) 403:26–36. doi: 10.1016/j.jim.2013.11.014 24287423

[50] Su Z Tao X. Current understanding of IL-37 in human health and disease. Front Immunol (2021) 12:696605. doi: 10.3389/fimmu.2021.696605 34248996 PMC8267878

[51] Brunt VE Ikoba AP Ziemba BP Ballak DB Hoischen A Dinarello CA. Circulating interleukin-37 declines with aging in healthy humans: relations to healthspan indicators and IL37 gene SNPs. Geroscience (2022) 27:1–20. doi: 10.1007/s11357-022-00587-3 PMC9137444 35622271

