# Supplementary material for: Circulating interleukin-38 concentrations in healthy adults
Source: Front Immunol. 2022 Aug 9;13:964365. doi: 10.3389/fimmu.2022.964365 (PMC9396651; doi:10.3389/fimmu.2022.964365)
Supplement: Supplementary file 1 [file DataSheet_1.docx]

Supplementary Material

# Supplementary Figures and Tables

**Supplementary Table 1 –** Significant eQTLs in *IL1F10* in single tissues obtained from GTEx

| **Chromosome** | **Start** | **End** | **Reference** | **Variant** | **SNP ID** | **P-value** |
| --- | --- | --- | --- | --- | --- | --- |
| chr2 | 113109711 | 113109711 | T | C | rs55709272 | 4,90E-12 |
| chr2 | 113083453 | 113083453 | A | G | rs6734238 | 8,50E-11 |
| chr2 | 113119202 | 113119202 | G | A | rs2637988 | 3,70E-10 |
| chr2 | 112974686 | 112974686 | A | G | rs11695148 | 4,30E-10 |
| chr2 | 113119446 | 113119446 | G | A | rs2592346 | 7,10E-10 |
| chr2 | 113089161 | 113089161 | A | G | rs12328766 | 8,00E-10 |
| chr2 | 113095879 | 113095879 | G | T | rs62158846 | 8,20E-10 |
| chr2 | 113126824 | 113126824 | G | A | rs439154 | 8,70E-10 |
| chr2 | 113095804 | 113095804 | A | G | rs9973741 | 1,10E-09 |
| chr2 | 113096072 | 113096072 | T | A | rs58865280 | 1,10E-09 |
| chr2 | 113096543 | 113096543 | T | A | rs6746979 | 1,10E-09 |
| chr2 | 113097266 | 113097266 | A | G | rs7596350 | 1,10E-09 |
| chr2 | 113102184 | 113102184 | G | A | rs7574159 | 1,10E-09 |
| chr2 | 113105959 | 113105959 | A | G | rs13382561 | 1,10E-09 |
| chr2 | 113128773 | 113128773 | A | G | rs1794066 | 1,20E-09 |
| chr2 | 113061757 | 113061757 | A | C | rs2515398 | 1,20E-09 |
| chr2 | 113061805 | 113061805 | C | T | rs6733859 | 1,20E-09 |
| chr2 | 113061842 | 113061842 | A | G | rs6748448 | 1,20E-09 |
| chr2 | 113061874 | 113061874 | G | T | rs2515399 | 1,20E-09 |
| chr2 | 113061959 | 113061959 | A | G | rs2472187 | 1,20E-09 |
| chr2 | 113062037 | 113062037 | T | C | rs2252007 | 1,20E-09 |
| chr2 | 113118636 | 113118636 | T | G | rs315919 | 2,00E-09 |
| chr2 | 113089430 | 113089430 | G | A | rs12329129 | 2,40E-09 |
| chr2 | 113129198 | 113129198 | T | C | rs1665190 | 2,70E-09 |
| chr2 | 113083938 | 113083938 | C | T | rs6722922 | 2,70E-09 |
| chr2 | 113084929 | 113084929 | T | C | rs13398728 | 2,70E-09 |
| chr2 | 113089567 | 113089567 | C | G | rs12328368 | 3,00E-09 |
| chr2 | 113092685 | 113092685 | C | G | rs7587033 | 3,00E-09 |
| chr2 | 113093215 | 113093215 | C | A | rs6738239 | 3,00E-09 |
| chr2 | 113093582 | 113093582 | A | T | rs1446509 | 3,00E-09 |
| chr2 | 113083955 | 113083955 | G | A | rs6750559 | 3,20E-09 |
| chr2 | 113086807 | 113086807 | G | A | rs6741180 | 3,20E-09 |
| chr2 | 113082866 | 113082866 | A | T | rs10188292 | 3,30E-09 |
| chr2 | 113085706 | 113085706 | G | A | rs13410964 | 3,30E-09 |
| chr2 | 113107837 | 113107837 | G | A | rs28648961 | 3,40E-09 |
| chr2 | 113106433 | 113106433 | A | C | rs17207494 | 3,40E-09 |
| chr2 | 113093940 | 113093940 | C | T | rs1446510 | 3,40E-09 |
| chr2 | 113082998 | 113082998 | C | G | rs10176274 | 3,60E-09 |
| chr2 | 113098058 | 113098058 | G | C | rs55896126 | 3,70E-09 |
| chr2 | 113081578 | 113081578 | T | C | rs1867830 | 4,20E-09 |
| chr2 | 113102461 | 113102461 | G | A | rs7574427 | 4,60E-09 |
| chr2 | 113059604 | 113059604 | C | T | rs2515392 | 4,80E-09 |
| chr2 | 113059691 | 113059691 | T | C | rs2441375 | 4,80E-09 |
| chr2 | 113059825 | 113059825 | G | A | rs1530548 | 4,80E-09 |
| chr2 | 113059936 | 113059936 | T | A | rs1530550 | 4,80E-09 |
| chr2 | 113059989 | 113059989 | T | C | rs1530551 | 4,80E-09 |
| chr2 | 113060141 | 113060141 | CT |  | rs3075085 | 4,80E-09 |
| chr2 | 113060178 | 113060178 | T | C | rs2441376 | 4,80E-09 |
| chr2 | 113060188 | 113060188 | C | T | rs2515394 | 4,80E-09 |
| chr2 | 113060543 | 113060543 | G | T | rs1530552 | 4,80E-09 |
| chr2 | 113060594 | 113060594 | G | A | rs1530553 | 4,80E-09 |
| chr2 | 113061055 | 113061055 | G | A | rs2251872 | 4,80E-09 |
| chr2 | 113061125 | 113061125 | G | C | rs2515395 | 4,80E-09 |
| chr2 | 113061161 | 113061161 | G | A | rs1374285 | 4,80E-09 |
| chr2 | 113061430 | 113061430 | C | G | rs1374286 | 4,80E-09 |
| chr2 | 113061575 | 113061575 |  | AT | rs10633611 | 4,80E-09 |
| chr2 | 113094987 | 113094987 | G | A | rs13424580 | 6,20E-09 |
| chr2 | 113109277 | 113109277 | G | T | rs7580634 | 6,40E-09 |
| chr2 | 113068898 | 113068898 | A | G | rs10206428 | 1,30E-08 |
| chr2 | 113097143 | 113097143 | G | T | rs2087705 | 1,40E-08 |
| chr2 | 113061598 | 113061598 | G | A | rs2515396 | 1,50E-08 |
| chr2 | 113091384 | 113091384 | C | T | rs6730516 | 1,60E-08 |
| chr2 | 113086898 | 113086898 | C | T | rs4496335 | 1,70E-08 |
| chr2 | 113063078 | 113063078 | A | T | rs3180234 | 2,00E-08 |
| chr2 | 113135197 | 113135197 | A | G | rs315949 | 2,80E-08 |
| chr2 | 113082481 | 113082481 | G | C | rs6743171 | 2,80E-08 |
| chr2 | 113062899 | 113062899 | T | C | rs2515401 | 2,80E-08 |
| chr2 | 113063095 | 113063095 | A | G | rs3180235 | 2,80E-08 |
| chr2 | 113063155 | 113063155 | T | C | rs2515403 | 2,80E-08 |
| chr2 | 113063237 | 113063237 | C | G | rs2472188 | 2,80E-08 |
| chr2 | 113063623 | 113063623 | T | C | rs957201 | 2,80E-08 |
| chr2 | 113064076 | 113064076 | T | C | rs768627 | 2,80E-08 |
| chr2 | 113066049 | 113066049 | T | C | rs12475161 | 2,80E-08 |
| chr2 | 113067263 | 113067263 | A | T | rs4849146 | 2,80E-08 |
| chr2 | 113091077 | 113091077 | C | T | rs4849153 | 3,30E-08 |
| chr2 | 113080525 | 113080525 | G | A | rs13409360 | 3,40E-08 |
| chr2 | 113080568 | 113080568 | G | A | rs13409371 | 3,40E-08 |
| chr2 | 113136761 | 113136761 | G | A | rs315943 | 3,50E-08 |
| chr2 | 113131484 | 113131484 | G | A | rs452204 | 3,60E-08 |
| chr2 | 113092537 | 113092537 | G | A | rs11123162 | 3,70E-08 |
| chr2 | 113058420 | 113058420 | C | T | rs2515391 | 3,90E-08 |
| chr2 | 113063003 | 113063003 | C | A | rs2515402 | 4,00E-08 |
| chr2 | 113062953 | 113062953 | A | G | rs1800930 | 4,50E-08 |
| chr2 | 113061150 | 113061150 | A | G | rs2251876 | 4,90E-08 |
| chr2 | 113083453 | 113083453 | A | G | rs6734238 | 5,00E-08 |
| chr2 | 113113148 | 113113148 | T | G | rs62158854 | 5,80E-08 |
| chr2 | 113116890 | 113116890 | T | C | rs4251961 | 6,00E-08 |
| chr2 | 113111989 | 113111989 | C | T | rs62158853 | 6,40E-08 |
| chr2 | 113112422 | 113112422 | T | C | rs315933 | 6,60E-08 |
| chr2 | 113113581 | 113113581 |  | AAT | rs55663133 | 7,90E-08 |
| chr2 | 113061862 | 113061862 | A | G | rs3820738 | 1,10E-07 |
| chr2 | 113109711 | 113109711 | T | C | rs55709272 | 1,30E-07 |
| chr2 | 113089161 | 113089161 | A | G | rs12328766 | 1,60E-07 |
| chr2 | 113146972 | 113146972 | C | T | rs6750555 | 2,20E-07 |
| chr2 | 113089430 | 113089430 | G | A | rs12329129 | 2,20E-07 |
| chr2 | 113086898 | 113086898 | C | T | rs4496335 | 2,30E-07 |
| chr2 | 113091384 | 113091384 | C | T | rs6730516 | 2,30E-07 |
| chr2 | 113094987 | 113094987 | G | A | rs13424580 | 2,40E-07 |
| chr2 | 113082866 | 113082866 | A | T | rs10188292 | 2,50E-07 |
| chr2 | 113085706 | 113085706 | G | A | rs13410964 | 2,50E-07 |
| chr2 | 113146253 | 113146253 | A | T | rs2172190 | 2,80E-07 |
| chr2 | 113148195 | 113148195 | G | A | rs2264098 | 2,80E-07 |
| chr2 | 113083938 | 113083938 | C | T | rs6722922 | 2,80E-07 |
| chr2 | 113084929 | 113084929 | T | C | rs13398728 | 2,80E-07 |
| chr2 | 113082998 | 113082998 | C | G | rs10176274 | 2,90E-07 |
| chr2 | 113098058 | 113098058 | G | C | rs55896126 | 2,90E-07 |
| chr2 | 113083955 | 113083955 | G | A | rs6750559 | 3,10E-07 |
| chr2 | 113086807 | 113086807 | G | A | rs6741180 | 3,10E-07 |
| chr2 | 113089567 | 113089567 | C | G | rs12328368 | 3,10E-07 |
| chr2 | 113092685 | 113092685 | C | G | rs7587033 | 3,10E-07 |
| chr2 | 113093215 | 113093215 | C | A | rs6738239 | 3,10E-07 |
| chr2 | 113093582 | 113093582 | A | T | rs1446509 | 3,10E-07 |
| chr2 | 113093940 | 113093940 | C | T | rs1446510 | 3,10E-07 |
| chr2 | 113106433 | 113106433 | A | C | rs17207494 | 3,60E-07 |
| chr2 | 113080263 | 113080263 | T | C | rs12711752 | 3,90E-07 |
| chr2 | 113137939 | 113137939 | C | A | rs2902452 | 4,20E-07 |
| chr2 | 113080525 | 113080525 | G | A | rs13409360 | 4,20E-07 |
| chr2 | 113080568 | 113080568 | G | A | rs13409371 | 4,20E-07 |
| chr2 | 113145477 | 113145477 | A | G | rs315956 | 4,50E-07 |
| chr2 | 113082481 | 113082481 | G | C | rs6743171 | 4,60E-07 |
| chr2 | 113142600 | 113142600 | A | G | rs11885498 | 4,70E-07 |
| chr2 | 113144613 | 113144613 | T | C | rs11123166 | 4,80E-07 |
| chr2 | 113144885 | 113144885 | T | C | rs62161285 | 4,80E-07 |
| chr2 | 113102461 | 113102461 | G | A | rs7574427 | 5,50E-07 |
| chr2 | 113095879 | 113095879 | G | T | rs62158846 | 6,00E-07 |
| chr2 | 113148147 | 113148147 | G | A | rs2264097 | 6,10E-07 |
| chr2 | 113107837 | 113107837 | G | A | rs28648961 | 6,30E-07 |
| chr2 | 113097952 | 113097952 | G | A | rs4575729 | 6,40E-07 |
| chr2 | 113103284 | 113103284 | C | A | rs10207930 | 6,40E-07 |
| chr2 | 113103818 | 113103818 | C | T | rs13432105 | 6,40E-07 |
| chr2 | 113103819 | 113103819 | A | C | rs13394316 | 6,40E-07 |
| chr2 | 113095804 | 113095804 | A | G | rs9973741 | 6,90E-07 |
| chr2 | 113096072 | 113096072 | T | A | rs58865280 | 6,90E-07 |
| chr2 | 113096543 | 113096543 | T | A | rs6746979 | 6,90E-07 |
| chr2 | 113097266 | 113097266 | A | G | rs7596350 | 6,90E-07 |
| chr2 | 113102184 | 113102184 | G | A | rs7574159 | 6,90E-07 |
| chr2 | 113105959 | 113105959 | A | G | rs13382561 | 6,90E-07 |
| chr2 | 113106194 | 113106194 | T | C | rs2029582 | 7,10E-07 |
| chr2 | 113106901 | 113106901 |  | AAA | rs11473501 | 7,10E-07 |
| chr2 | 113141212 | 113141212 | C | G | rs1374281 | 8,80E-07 |
| chr2 | 113071001 | 113071001 | G | A | rs7570267 | 1,00E-06 |
| chr2 | 113141448 | 113141448 | T | C | rs895496 | 1,00E-06 |
| chr2 | 113084205 | 113084205 | T | A | rs11687782 | 1,10E-06 |
| chr2 | 113086459 | 113086459 | C | T | rs7561598 | 1,10E-06 |
| chr2 | 113086976 | 113086976 | T | C | rs6731551 | 1,10E-06 |
| chr2 | 113109277 | 113109277 | G | T | rs7580634 | 1,10E-06 |
| chr2 | 113092260 | 113092260 | C | T | rs1542176 | 1,20E-06 |
| chr2 | 113093325 | 113093325 | C | G | rs6738377 | 1,20E-06 |
| chr2 | 113087023 | 113087023 | A | G | rs6728590 | 1,50E-06 |
| chr2 | 113087503 | 113087503 | A | T | rs4368340 | 1,50E-06 |
| chr2 | 113088001 | 113088001 | C | T | rs11123161 | 1,50E-06 |
| chr2 | 113050794 | 113050794 | G | A | rs12995447 | 1,70E-06 |
| chr2 | 113055537 | 113055537 | C | T | rs10165821 | 1,70E-06 |
| chr2 | 113051410 | 113051410 | G | A | rs11687093 | 1,70E-06 |
| chr2 | 113065995 | 113065995 | A | G | rs11898158 | 1,70E-06 |
| chr2 | 113033616 | 113033616 | C | T | rs6758965 | 1,90E-06 |
| chr2 | 113149292 | 113149292 | C | G | rs11123167 | 2,10E-06 |
| chr2 | 113053823 | 113053823 | T | C | rs11687630 | 2,70E-06 |
| chr2 | 113074735 | 113074735 | T | C | rs6761276 | 2,90E-06 |
| chr2 | 113043796 | 113043796 | C | T | rs10210878 | 3,10E-06 |
| chr2 | 113044321 | 113044321 | T | C | rs2034260 | 3,10E-06 |
| chr2 | 113068389 | 113068389 | T | C | rs921065 | 3,20E-06 |
| chr2 | 113080188 | 113080188 | T | G | rs12711751 | 3,50E-06 |
| chr2 | 113034486 | 113034486 |  | TATT | rs58701438 | 3,70E-06 |
| chr2 | 113086533 | 113086533 | A | G | rs7575402 | 4,60E-06 |
| chr2 | 113034473 | 113034473 | T | C | rs60078854 | 4,70E-06 |
| chr2 | 113114412 | 113114412 | T |  | rs10712923 | 5,10E-06 |
| chr2 | 113039089 | 113039089 | C | T | rs1374280 | 5,50E-06 |
| chr2 | 113081031 | 113081031 | A | G | rs10181720 | 6,10E-06 |
| chr2 | 113081051 | 113081051 | T | C | rs10184259 | 6,10E-06 |
| chr2 | 113081075 | 113081075 | C | T | rs10169599 | 6,10E-06 |
| chr2 | 113037182 | 113037182 | C | T | rs13407508 | 7,20E-06 |
| chr2 | 113081578 | 113081578 | T | C | rs1867830 | 7,30E-06 |
| chr2 | 113145477 | 113145477 | A | G | rs315956 | 7,80E-06 |
| chr2 | 113148147 | 113148147 | G | A | rs2264097 | 7,80E-06 |
| chr2 | 113148195 | 113148195 | G | A | rs2264098 | 7,80E-06 |
| chr2 | 113019813 | 113019813 | T | A | rs35002769 | 8,10E-06 |
| chr2 | 113069182 | 113069182 | C | T | rs2862853 | 8,10E-06 |
| chr2 | 113069221 | 113069221 | T | C | rs2862854 | 8,10E-06 |
| chr2 | 113069222 | 113069222 | G | A | rs2862855 | 8,10E-06 |
| chr2 | 113069250 | 113069250 | C | T | rs1867832 | 8,10E-06 |
| chr2 | 113069255 | 113069255 | A | G | rs1867833 | 8,10E-06 |
| chr2 | 113057149 | 113057149 |  | T | rs28928313 | 9,50E-06 |
| chr2 | 113053370 | 113053370 | T | A | rs9678578 | 1,00E-05 |
| chr2 | 113054859 | 113054859 | A | C | rs6542110 | 1,00E-05 |
| chr2 | 113055938 | 113055938 | G | C | rs1813048 | 1,00E-05 |
| chr2 | 113057870 | 113057870 | C | T | rs2515400 | 1,00E-05 |
| chr2 | 113058513 | 113058513 | T | A | rs2441374 | 1,00E-05 |
| chr2 | 113063540 | 113063540 | C | T | rs2515404 | 1,00E-05 |
| chr2 | 113067508 | 113067508 | T | C | rs7599662 | 1,00E-05 |
| chr2 | 113067809 | 113067809 | C | A | rs2100071 | 1,00E-05 |
| chr2 | 113069303 | 113069303 | A |  | rs5833479 | 1,00E-05 |
| chr2 | 113146253 | 113146253 | A | T | rs2172190 | 1,00E-05 |
| chr2 | 113084205 | 113084205 | T | A | rs11687782 | 1,10E-05 |
| chr2 | 113086459 | 113086459 | C | T | rs7561598 | 1,10E-05 |
| chr2 | 113086976 | 113086976 | T | C | rs6731551 | 1,10E-05 |
| chr2 | 113077243 | 113077243 | A | C | rs13386602 | 1,20E-05 |
| chr2 | 113077258 | 113077258 | G | A | rs13398125 | 1,20E-05 |
| chr2 | 113077308 | 113077308 | T | C | rs13389457 | 1,20E-05 |
| chr2 | 113077734 | 113077734 | T | A | rs11887823 | 1,20E-05 |
| chr2 | 113077742 | 113077742 | G | A | rs11891557 | 1,20E-05 |
| chr2 | 113077803 | 113077803 | C | A | rs12711750 | 1,20E-05 |
| chr2 | 113077943 | 113077943 | A | G | rs11677043 | 1,20E-05 |
| chr2 | 113077945 | 113077945 | G | A | rs11682107 | 1,20E-05 |
| chr2 | 113078025 | 113078025 | C | T | rs11693750 | 1,20E-05 |
| chr2 | 113078026 | 113078026 | A | G | rs11677088 | 1,20E-05 |
| chr2 | 113078114 | 113078114 | T | C | rs11678375 | 1,20E-05 |
| chr2 | 113078331 | 113078331 | C | T | rs12477866 | 1,20E-05 |
| chr2 | 113078342 | 113078342 | C | A | rs12477867 | 1,20E-05 |
| chr2 | 113078422 | 113078422 | T | C | rs12466799 | 1,20E-05 |
| chr2 | 113078505 | 113078505 | AT |  | rs35405134 | 1,20E-05 |
| chr2 | 113078771 | 113078771 | C | T | rs6759676 | 1,20E-05 |
| chr2 | 113078949 | 113078949 |  | G | rs34124861 | 1,20E-05 |
| chr2 | 113079263 | 113079263 | A |  | rs35997925 | 1,20E-05 |
| chr2 | 113050934 | 113050934 | T | C | rs6728769 | 1,20E-05 |
| chr2 | 113126824 | 113126824 | G | A | rs439154 | 1,20E-05 |
| chr2 | 113046807 | 113046807 |  | TTGA | rs33976060 | 1,30E-05 |
| chr2 | 113076855 | 113076855 | G |  | rs61094379 | 1,30E-05 |
| chr2 | 113058904 | 113058904 | C | T | rs990524 | 1,30E-05 |
| chr2 | 113029779 | 113029779 | G | C | rs4849143 | 1,40E-05 |
| chr2 | 113092260 | 113092260 | C | T | rs1542176 | 1,50E-05 |
| chr2 | 113093325 | 113093325 | C | G | rs6738377 | 1,50E-05 |
| chr2 | 113087023 | 113087023 | A | G | rs6728590 | 1,60E-05 |
| chr2 | 113087503 | 113087503 | A | T | rs4368340 | 1,60E-05 |
| chr2 | 113088001 | 113088001 | C | T | rs11123161 | 1,60E-05 |
| chr2 | 113113581 | 113113581 |  | AAT | rs55663133 | 1,60E-05 |
| chr2 | 113135197 | 113135197 | A | G | rs315949 | 1,60E-05 |
| chr2 | 113128773 | 113128773 | A | G | rs1794066 | 2,00E-05 |
| chr2 | 113149292 | 113149292 | C | G | rs11123167 | 2,10E-05 |
| chr2 | 113103818 | 113103818 | C | T | rs13432105 | 2,20E-05 |
| chr2 | 113103819 | 113103819 | A | C | rs13394316 | 2,20E-05 |
| chr2 | 113106194 | 113106194 | T | C | rs2029582 | 2,30E-05 |
| chr2 | 113106901 | 113106901 |  | AAA | rs11473501 | 2,30E-05 |
| chr2 | 113120136 | 113120136 | A | C | rs878972 | 2,30E-05 |
| chr2 | 113097952 | 113097952 | G | A | rs4575729 | 2,40E-05 |
| chr2 | 113103284 | 113103284 | C | A | rs10207930 | 2,40E-05 |
| chr2 | 113065574 | 113065574 | T | G | rs17042750 | 2,40E-05 |
| chr2 | 113068646 | 113068646 | C | G | rs28928270 | 2,40E-05 |
| chr2 | 113113148 | 113113148 | T | G | rs62158854 | 2,50E-05 |
| chr2 | 113129198 | 113129198 | T | C | rs1665190 | 2,50E-05 |
| chr2 | 113141448 | 113141448 | T | C | rs895496 | 2,60E-05 |
| chr2 | 113072292 | 113072292 | G | T | rs13019891 | 2,60E-05 |
| chr2 | 113111977 | 113111977 | C | A | rs315930 | 2,60E-05 |
| chr2 | 113112226 | 113112226 | C | T | rs1630153 | 2,60E-05 |
| chr2 | 113112266 | 113112266 | C | A | rs315931 | 2,60E-05 |
| chr2 | 113108792 | 113108792 | C | T | rs315927 | 2,90E-05 |
| chr2 | 113117391 | 113117391 | G | C | rs4251967 | 2,90E-05 |
| chr2 | 113117504 | 113117504 | C | T | rs11677397 | 2,90E-05 |
| chr2 | 113112400 | 113112400 | T | C | rs315932 | 3,00E-05 |
| chr2 | 113117640 | 113117640 | A | C | rs4251968 | 3,00E-05 |
| chr2 | 113117851 | 113117851 | G | A | rs2234676 | 3,00E-05 |
| chr2 | 113117932 | 113117932 | G | A | rs2234677 | 3,00E-05 |
| chr2 | 113117988 | 113117988 | A | G | rs2234678 | 3,00E-05 |
| chr2 | 113118007 | 113118007 | G | C | rs2234679 | 3,00E-05 |
| chr2 | 113118054 | 113118054 | T | C | rs16065 | 3,00E-05 |
| chr2 | 113118176 | 113118176 | C | G | rs4251969 | 3,00E-05 |
| chr2 | 113118196 | 113118196 | A | G | rs4251970 | 3,00E-05 |
| chr2 | 113118501 | 113118501 | C | G | rs4251974 | 3,00E-05 |
| chr2 | 113118583 | 113118583 | A | G | rs4251975 | 3,00E-05 |
| chr2 | 113118603 | 113118603 | C | A | rs4251976 | 3,00E-05 |
| chr2 | 113118848 | 113118848 | C | T | rs4251977 | 3,00E-05 |
| chr2 | 113118911 | 113118911 | T | C | rs4251978 | 3,00E-05 |
| chr2 | 113118921 | 113118921 | T | C | rs4251979 | 3,00E-05 |
| chr2 | 113118962 | 113118962 | G | A | rs4251980 | 3,00E-05 |
| chr2 | 113119125 | 113119125 | G | A | rs4251981 | 3,00E-05 |
| chr2 | 113119442 | 113119442 | A | G | rs4251983 | 3,00E-05 |
| chr2 | 113119649 | 113119649 | A | G | rs4251984 | 3,00E-05 |
| chr2 | 113119836 | 113119836 | G | T | rs4251985 | 3,00E-05 |
| chr2 | 113119961 | 113119961 | G | C | rs4251986 | 3,00E-05 |
| chr2 | 113111989 | 113111989 | C | T | rs62158853 | 3,00E-05 |
| chr2 | 113005886 | 113005886 | A | C | rs2305152 | 3,10E-05 |
| chr2 | 113010010 | 113010010 | T | C | rs725140 | 3,10E-05 |
| chr2 | 112958600 | 112958600 | C | T | rs1317913 | 3,80E-05 |
| chr2 | 113070522 | 113070522 | C | T | rs1867834 | 3,80E-05 |
| chr2 | 113059363 | 113059363 | C | G | rs2278716 | 4,00E-05 |
| chr2 | 113059365 | 113059365 | A | C | rs2278717 | 4,00E-05 |
| chr2 | 113119202 | 113119202 | G | A | rs2637988 | 4,00E-05 |
| chr2 | 113128472 | 113128472 | G | A | rs3181052 | 4,00E-05 |
| chr2 | 113070873 | 113070873 | A | C | rs10165797 | 4,10E-05 |
| chr2 | 113069819 | 113069819 | G | A | rs1446521 | 4,20E-05 |
| chr2 | 113071659 | 113071659 | A | T | rs4849147 | 4,50E-05 |
| chr2 | 113116890 | 113116890 | T | C | rs4251961 | 4,80E-05 |
| chr2 | 112980835 | 112980835 | T | G | rs11683399 | 4,80E-05 |
| chr2 | 113136761 | 113136761 | G | A | rs315943 | 4,80E-05 |
| chr2 | 113086533 | 113086533 | A | G | rs7575402 | 4,80E-05 |
| chr2 | 113122156 | 113122156 | G | A | rs1794065 | 4,80E-05 |
| chr2 | 113119446 | 113119446 | G | A | rs2592346 | 5,00E-05 |
| chr2 | 113011237 | 113011237 | C | T | rs28946269 | 5,00E-05 |
| chr2 | 113131484 | 113131484 | G | A | rs452204 | 5,80E-05 |
| chr2 | 113131216 | 113131216 | A | T | rs454078 | 5,90E-05 |
| chr2 | 112954831 | 112954831 | G | A | rs11685257 | 6,00E-05 |
| chr2 | 113037620 | 113037620 | A | G | rs4848312 | 6,20E-05 |
| chr2 | 113092537 | 113092537 | G | A | rs11123162 | 6,40E-05 |
| chr2 | 113041578 | 113041578 |  | A | rs11390487 | 6,40E-05 |
| chr2 | 113111830 | 113111830 | T | C | rs315929 | 6,40E-05 |
| chr2 | 112923665 | 112923665 | A | C | rs75396415 | 6,50E-05 |
| chr2 | 113118636 | 113118636 | T | G | rs315919 | 6,90E-05 |
| chr2 | 113037600 | 113037600 | A | G | rs4848311 | 7,20E-05 |
| chr2 | 112999200 | 112999200 | T | G | rs13033104 | 8,10E-05 |
| chr2 | 113039988 | 113039988 | G | A | rs1900287 | 8,10E-05 |
| chr2 | 113044122 | 113044122 | G | A | rs2121333 | 8,10E-05 |
| chr2 | 113081709 | 113081709 | G | A | rs4848314 | 8,20E-05 |
| chr2 | 113084391 | 113084391 | T | C | rs55932259 | 8,20E-05 |
| chr2 | 113087928 | 113087928 | G | T | rs66613854 | 8,20E-05 |
| chr2 | 113089660 | 113089660 | G | C | rs66615594 | 8,20E-05 |
| chr2 | 113090546 | 113090546 | A | G | rs57130564 | 8,20E-05 |
| chr2 | 113092409 | 113092409 | A | C | rs12475887 | 8,20E-05 |
| chr2 | 113095773 | 113095773 | C | T | rs1665189 | 8,20E-05 |
| chr2 | 113098197 | 113098197 | A | T | rs1618084 | 8,20E-05 |
| chr2 | 113100105 | 113100105 | A | G | rs13032281 | 8,20E-05 |
| chr2 | 113100619 | 113100619 | C | A | rs1688075 | 8,20E-05 |
| chr2 | 113100918 | 113100918 | G | T | rs1688076 | 8,20E-05 |
| chr2 | 113102619 | 113102619 | T | C | rs1794071 | 8,20E-05 |
| chr2 | 113107790 | 113107790 | T | C | rs315925 | 8,20E-05 |
| chr2 | 113091077 | 113091077 | C | T | rs4849153 | 8,40E-05 |
| chr2 | 113053480 | 113053480 | A | G | rs11123158 | 8,60E-05 |
| chr2 | 113130557 | 113130557 | C | A | rs598859 | 8,80E-05 |
| chr2 | 113039684 | 113039684 | T | G | rs1446508 | 9,30E-05 |
| chr2 | 113075404 | 113075404 | C | T | rs13005572 | 1,00E-04 |
| chr2 | 113097143 | 113097143 | G | T | rs2087705 | 1,10E-04 |
| chr2 | 113130888 | 113130888 | A | G | rs448341 | 1,10E-04 |
| chr2 | 113130893 | 113130893 | C | G | rs434792 | 1,10E-04 |
| chr2 | 113032064 | 113032064 | G | A | rs6724667 | 1,10E-04 |
| chr2 | 113128249 | 113128249 | G | A | rs444413 | 1,20E-04 |
| chr2 | 113128308 | 113128308 |  | AG | rs4252008 | 1,20E-04 |
| chr2 | 113128926 | 113128926 | G | A | rs1794068 | 1,20E-04 |
| chr2 | 113129630 | 113129630 | T | C | rs419598 | 1,20E-04 |
| chr2 | 113129685 | 113129685 | C | T | rs423904 | 1,20E-04 |
| chr2 | 113129696 | 113129696 | G | A | rs446433 | 1,20E-04 |
| chr2 | 113129717 | 113129717 | G | C | rs495282 | 1,20E-04 |
| chr2 | 113129761 | 113129761 | A | C | rs495410 | 1,20E-04 |
| chr2 | 113129822 | 113129822 | G | A | rs442710 | 1,20E-04 |
| chr2 | 113129881 | 113129881 | G | T | rs408392 | 1,20E-04 |
| chr2 | 113130095 | 113130095 | A | G | rs447713 | 1,20E-04 |
| chr2 | 113130226 | 113130226 | A | G | rs128964 | 1,20E-04 |
| chr2 | 113075993 | 113075993 | C | T | rs13011842 | 1,20E-04 |
| chr2 | 113039867 | 113039867 | G | A | rs2862773 | 1,30E-04 |
| chr2 | 113039869 | 113039869 |  | GGGGT | rs5833478 | 1,30E-04 |
| chr2 | 113040068 | 113040068 | A | G | rs1900288 | 1,30E-04 |
| chr2 | 113040185 | 113040185 | C | T | rs2862775 | 1,30E-04 |
| chr2 | 113040298 | 113040298 | T | A | rs1900289 | 1,30E-04 |
| chr2 | 113040347 | 113040347 | G | A | rs2862776 | 1,30E-04 |
| chr2 | 113040391 | 113040391 | T | G | rs7557928 | 1,30E-04 |
| chr2 | 113040439 | 113040439 | G | A | rs7567108 | 1,30E-04 |
| chr2 | 113040715 | 113040715 | G | A | rs7570058 | 1,30E-04 |
| chr2 | 113040811 | 113040811 | G | A | rs7570159 | 1,30E-04 |
| chr2 | 113041082 | 113041082 | G | A | rs7570483 | 1,30E-04 |
| chr2 | 113042104 | 113042104 | G | C | rs1530554 | 1,30E-04 |
| chr2 | 113042359 | 113042359 |  | ATATTT | rs34868654 | 1,30E-04 |
| chr2 | 113042472 | 113042472 | A | G | rs4849144 | 1,30E-04 |
| chr2 | 113076855 | 113076855 | TG |  | rs61094379 | 1,30E-04 |
| chr2 | 113112422 | 113112422 | T | C | rs315933 | 1,30E-04 |
| chr2 | 113032550 | 113032550 | C | A | rs17042721 | 1,30E-04 |
| chr2 | 113033301 | 113033301 | G | T | rs34200521 | 1,30E-04 |
| chr2 | 113034594 | 113034594 | C | T | rs11883847 | 1,30E-04 |
| chr2 | 113131002 | 113131002 | T | C | rs432014 | 1,30E-04 |
| chr2 | 113156735 | 113156735 | G | A | rs6739883 | 1,40E-04 |
| chr2 | 113074756 | 113074756 | C | A | rs6743376 | 1,40E-04 |
| chr2 | 113030045 | 113030045 | A | G | rs35391805 | 1,40E-04 |
| chr2 | 112901133 | 112901133 | G | T | rs113465881 | 1,40E-04 |
| chr2 | 113126618 | 113126618 | T | C | rs392503 | 1,50E-04 |
| chr2 | 112819149 | 112819149 | C | G | rs35145107 | 1,50E-04 |
| chr2 | 113129906 | 113129906 | C | T | rs2071459 | 1,50E-04 |
| chr2 | 113051689 | 113051689 | C | T | rs11887249 | 1,60E-04 |
| chr2 | 113053159 | 113053159 | T | A | rs1992763 | 1,60E-04 |
| chr2 | 112827473 | 112827473 | C | T | rs12992780 | 1,60E-04 |
| chr2 | 113033531 | 113033531 | A | G | rs12614012 | 1,70E-04 |
| chr2 | 113043573 | 113043573 | T | A | rs10175899 | 1,70E-04 |
| chr2 | 113100149 | 113100149 | C | T | rs112534387 | 1,80E-04 |
| chr2 | 113101280 | 113101280 | G | A | rs34720511 | 1,80E-04 |
| chr2 | 113102342 | 113102342 | C | T | rs35998927 | 1,80E-04 |
| chr2 | 113045986 | 113045986 | G | A | rs7569284 | 1,90E-04 |
| chr2 | 113130980 | 113130980 | G | A | rs451578 | 1,90E-04 |
| chr2 | 113128807 | 113128807 | A | G | rs1794067 | 1,90E-04 |
| chr2 | 112969003 | 112969003 | T | A | rs35880601 | 2,00E-04 |
| chr2 | 113033310 | 113033310 | A | C | rs2218557 | 2,00E-04 |
| chr2 | 113126106 | 113126106 | A | G | rs4252001 | 2,10E-04 |
| chr2 | 113122715 | 113122715 | C | G | rs2853628 | 2,10E-04 |
| chr2 | 113052836 | 113052836 | G | A | rs1992762 | 2,10E-04 |
| chr2 | 113121720 | 113121720 | G | A | rs3213448 | 2,10E-04 |
| chr2 | 113036856 | 113036856 | A | G | rs12475423 | 2,20E-04 |
| chr2 | 113073382 | 113073382 | A | T | rs28928294 | 2,40E-04 |
| chr2 | 113122472 | 113122472 | T | G | rs4251991 | 2,40E-04 |
| chr2 | 113122916 | 113122916 | G | A | rs4251993 | 2,40E-04 |
| chr2 | 113126625 | 113126625 | G | C | rs3087262 | 2,40E-04 |
| chr2 | 113050477 | 113050477 | A | T | rs34466405 | 2,50E-04 |
| chr2 | 113051713 | 113051713 | G | A | rs11897709 | 2,50E-04 |
| chr2 | 113052709 | 113052709 | G | A | rs1992761 | 2,50E-04 |
| chr2 | 113099828 | 113099828 | G | A | rs111974309 | 2,70E-04 |
| chr2 | 113050995 | 113050995 | C | G | rs34517213 | 2,80E-04 |
| chr2 | 113137502 | 113137502 | G | A | rs74667587 | 3,00E-04 |
| chr2 | 112729303 | 112729303 | A | G | rs112903399 | 3,10E-04 |

**Supplementary Table 2 –** Significant eQTLs in the exon region of *IL1F10* with allele frequency data

| **rsIDs** | **Reference** | **Alternate** | **Frequency South Asian** | **Frequency East Asian** | **Frequency non-Finnish European** | **Protein effect** | **Gene component** |
| --- | --- | --- | --- | --- | --- | --- | --- |
| rs6743376 | C | A | 79,32 | 69,53 | 65,08 | missense variant | exon region |
| rs6761276 | T | C | 68,93 | 16,93 | 57,92 | missense variant | exon region |


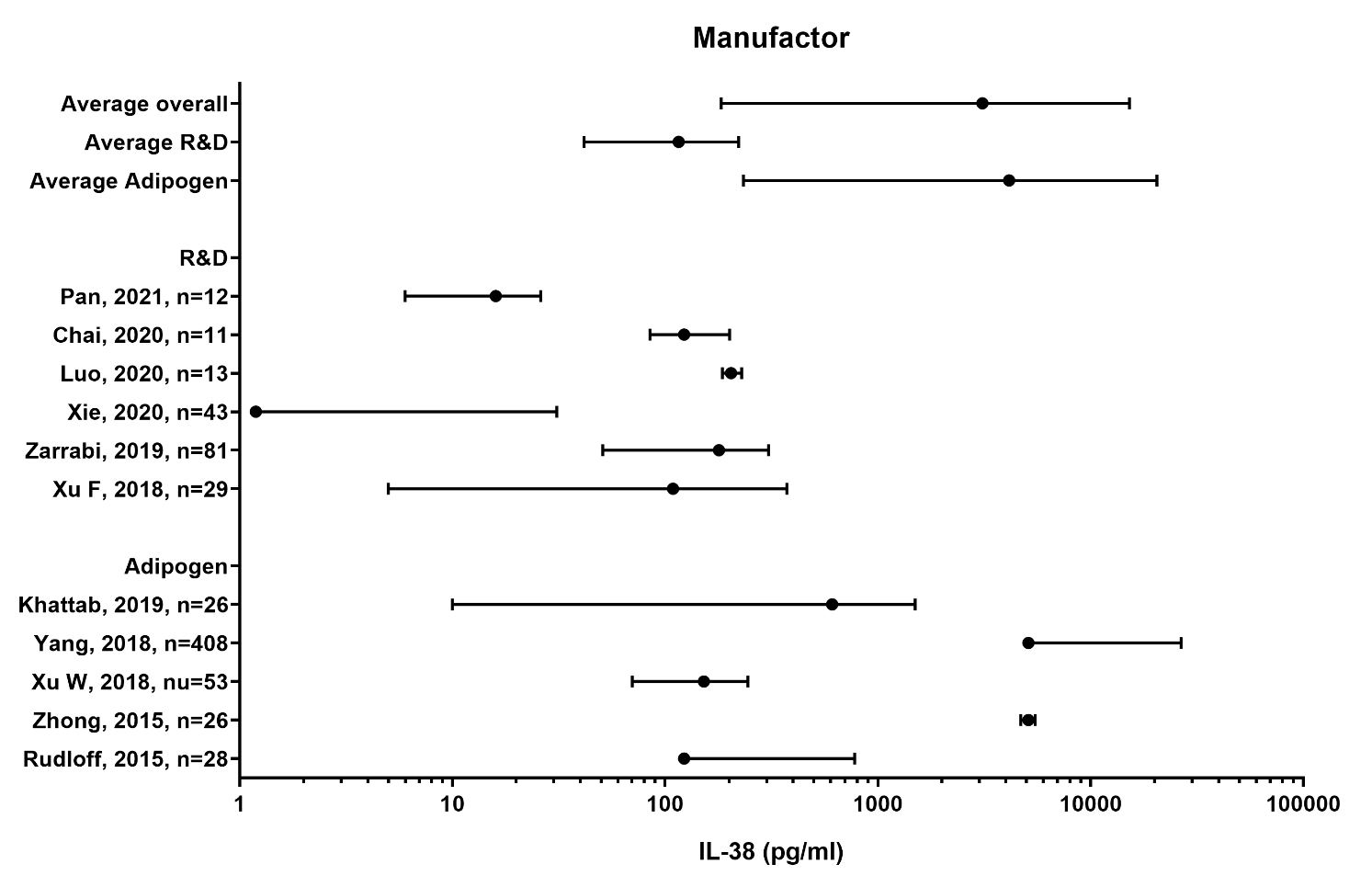


**Supplementary Figure 1.** Circulating IL-38 concentrations between studies stratified by most commonly used ELISA kits.
